# Anti‐TSNARE1 IgG plasma levels differ by sex in patients with schizophrenia in a Chinese population

**DOI:** 10.1002/2211-5463.12704

**Published:** 2019-09-04

**Authors:** Chan Li, Ruth Whelan, Hua Yang, Yaling Jiang, Chaosen Qiu, Qingyong Meng, Jun Wei

**Affiliations:** ^1^ Laboratory for Nursing Science & Institute of Laboratory Medicine Guangdong Medical University Dongguan China; ^2^ Institute of Health Research & Innovation University of the Highlands & Islands Inverness UK; ^3^ The Third People's Hospital of Jiangmen China

**Keywords:** autoantibodies, B lymphocytes, ELISA, gender difference, schizophrenia, TSNARE1

## Abstract

It was recently reported that levels of plasma IgG antibodies against peptide antigens derived from proteins encoded by schizophrenia‐associated genes are altered in individuals with schizophrenia treated with antipsychotics. This study aimed to replicate the initial finding in antipsychotic‐naïve patients with first‐episode schizophrenia and to explore the possible mechanism by which immune tolerance of B cells may be altered in this disease. A total of 408 case–control plasma samples were collected for analysis of circulating IgG antibodies against fragments derived from TCF4, TSNARE1, ZNF804A, TRANK1, ERCC4, DPYD and CD25 using an in‐house ELISA. The Mann–Whitney *U*‐test revealed that patients with schizophrenia had a significant change in plasma anti‐TSNARE1 and anti‐CD25 IgG levels; male patients mainly contributed to the increased levels of anti‐TSNARE1 IgG and anti‐CD25 IgG. Receiver operating characteristic (ROC) curve analysis revealed that the anti‐TSNARE1 IgG assay had an area under the ROC curve of 0.625 with a sensitivity of 15.7% and a specificity of 95.2%. Work on a B‐cell model revealed that TRANK1‐derived antigen treatments could enhance the proportions of CD83+ cells and apoptotic B cells when compared with TSNARE1‐derived antigen and vehicle treatment. We conclude that there is a gender difference in autoimmune responses in schizophrenia and suggest that anti‐TSNARE1 IgG may be indicative of schizophrenia in a subgroup of male patients.

**Abbreviations**
APC: antigen‐presenting cells
AUC: area under the ROC curve
CD: cluster of differentiation
CSF: cerebrospinal fluid
CXCR5: C‐X‐C chemokine receptor type 5
DPYD: dihydropyrimidine dehydrogenase
DRD2: dopamine receptor 2
ERCC4: ERCC excision repair 4, endonuclease catalytic subunit
FACS: fluorescence‐activated cell sorting
IgG: immunoglobulin G
IL: interleukin
MAD1L1: mitotic arrest deficient 1 like 1
MMP16: matrix metallopeptidase 16
NC: negative control
OD: optical density
PC: positive control
ROC: receiver operating characteristic
SBR: specific binding ratio
SD: standard deviation
SE: standard error
TCF4: transcription factor 4
TRANK1: tetratricopeptide repeat and ankyrin repeat containing 1
TSNARE1: t‐SNARE domain containing 1
VRK2: VRK serine/threonine kinase 2
ZNF804A: zinc finger protein 804A

Schizophrenia is a severe mental health condition, affecting around 0.7% of the population worldwide, and has been ranked among the top 10 causes of disability for both men and women 1. A definitive aetiology for the development of schizophrenia remains unclear although disturbances of the dopamine and glutamate neurotransmitter systems in the brain have been identified in the pathophysiology of the disease. It is possible that due to the vastly differing clinical presentation, schizophrenia is actually an umbrella term for a number of mechanistically distinct disorders. The identification of a pathological pathway for schizophrenia may lead to a greater understanding and personalised treatment of the disease, potentially addressing the issue of treatment of antipsychotic‐resistant schizophrenia.

Bechter *et al*. 2 reported that in a group of 63 hospitalised nonresponsive schizophrenia patients, 41% demonstrated abnormalities of immunological/inflammatory components in the cerebrospinal fluid (CSF). Steiner *et al*. 3 identified that individuals with first‐episode schizophrenia had reduced number of T helper cells and increased number of B lymphocytes, which returned to normal following medication. A further study found medicated individuals with schizophrenia had increased number of naïve B cells and CXCR5+ memory T cells, and decreased number of CD4+ memory T cells 4. Epidemiological evidence from national registries has described a cohort of individuals with genetic vulnerabilities for immune disorders and psychosis 5, 6. Both pro‐ and anti‐inflammatory mediators show differences between individuals with first‐episode psychosis, individuals on antipsychotic medication and healthy controls 7, 8. Interleukin‐10 (IL‐10) levels were found to be correlated with improvement in symptoms 9; meta‐analysis conducted by Gallego *et al*. 10 demonstrated an increase in CSF levels of IL‐8, IL‐1β and IL‐6 in patients with schizophrenia, further substantiated by Romeo *et al*. 11 who investigated these pro‐inflammatory cytokine levels in blood.

Severe infections and autoimmune disorders over a lifetime appear to confer additive risk of schizophrenia and schizophrenia spectrum disorders 5. Drug‐naïve individuals with first‐episode psychosis have shown an increased level of serum antibodies against neuronal cell surface molecules 12. Schizophrenia‐related changes in immunoglobulin levels in both plasma and CSF support an immunological role in the pathogenesis of the disease 13. A review by Goldsmith and Rogers 14 demonstrated contrasting reports of autoantibodies, due to heterogeneity between studies and failure to account for the confounding effects of medication.

Schizophrenia is a highly heritable disease with complex modes of transmission. A recent genome‐wide association study with meta‐analysis has confirmed 108 genetic loci significantly associated with risk of the disease 15. The candidate genes present in most loci were found to be highly expressed in both brain tissues and B lymphocytes (CD20+ and CD19+ cells), based on enrichment analysis of over 50 human tissues and cell lines, supporting the hypothesis that humoral immunity is likely to be involved in developing the disease 15. Further support of an immunological role in schizophrenia is the genetic association for the human leucocyte antigen region in the short arm of chromosome 6 15. It is possible that some proteins encoded by schizophrenia‐associated genes could trigger autoimmune responses. In a recent study, we identified nine novel autoantibodies (against DPYD, MAD1L1, ZNF804A, DRD2, TRANK1, VRK2, TCF4, TSNARE1, ERCC4 and MMP16) that were strongly associated with schizophrenia, irrespective of medication prescribed to patients with the disease 16. Accordingly, the present study was designed to investigate a potential mechanism by which circulating levels of IgG antibodies against linear peptide antigens could be significantly altered in schizophrenia based on our previous study 16. To explore how autoantigens of interest interact with B lymphocytes, a B‐cell model was developed with examination of apoptosis and B‐cell activation marker CD83.

## Materials and methods

### Study samples

A total of 214 patients with first‐episode psychosis (107 males and 107 females) were recruited for this study in the period between January and December 2017 by the Third People's Hospital of Jiangmen, Jiangmen, China. Of these 214 patients who all were drug‐naïve at the time of taking blood samples, 181 were confirmed as having schizophrenia using the International Statistical Classification of Diseases and Related Health Problems, 10th Revision after antipsychotic medication at least for 3 months, and 33 whose diagnosis was changed after antipsychotic medication were excluded from this study. The patients were drug‐naïve for any antipsychotic medication. Meanwhile, 227 healthy controls (122 males and 105 females) were recruited from local communities during the same period; eligible control subjects did not have previous or current diagnoses of mental health or neurological conditions. The participants who had history of either an autoimmune condition or a malignant disease were excluded from this study. All subjects were of the Chinese Han origin, and patients or a close relative such as parents gave written informed consent to participate in this study. It is worth mentioning that most participants had been included in a previous study 17.

A 5 mL volume of blood samples was collected from each participant for separation of plasma, and all blood samples were collected from patients just before they received antipsychotic medication. All the samples were taken in the morning, and plasma was separated by centrifugation at 2000 ***g*** at 4 °C for 10 min. All plasma samples were kept at −20 °C for less than a week and then stored at −80 °C in a laboratory based at Guangdong Medical University until use.

This study was approved by the Ethics Committee of Guangdong Medical University and conformed to the provisions of the Declaration of Helsinki.

### Detection of IgG antibodies against peptide antigens

An ELISA was developed in‐house to detect plasma IgG antibodies against target proteins as reported by our recent study, in which six linear peptide antigens of interest were selected for this study (Table S1), as described in our previous report 16. In addition, plasma IgG against IL‐2 receptor subunit alpha, also called CD25, was measured to explore the mechanism by which autoimmune response may be changed in schizophrenia.

All peptide antigens were synthesised by solid‐phase chemistry with a purity of > 95%. Each peptide antigen was dissolved in 67% acetic acid to a concentration of 5 mg·mL^−1^ and diluted in coating buffer (0.1 m phosphate buffer containing 0.15 m NaCl and 10 mm EDTA, pH 7.2) to the working solution of 20 μg·mL^−1^. Based on our previous reports, maleimide‐activated 96‐well plates (Thermo Fisher Scientific, Shanghai, China) were coated with 100 μL of antigen working solution and incubated overnight at 4 °C 16, 17, 18. Antigen‐coated plates, once dried, were sealed with sealing film and stored at 4 °C until use. The sealing film was removed just before use, and the plates were washed twice with 200 μL of wash buffer (PBS, containing 0.1% Tween‐20). The plasma sample (including positive control, PC) was diluted 1 : 150 in assay buffer (PBS containing 0.5% bovine serum albumin), and 50 μL of the sample was loaded into each sample well; 50 μL of assay buffer was added to each negative control (NC) well. Following incubation at room temperature for 90 min, the plate was washed three times with 200 μL of wash buffer and 50 μL of peroxidase‐conjugated goat anti‐human IgG Fc (ab98624; Abcam, Guangzhou, China) diluted 1 : 50 000 in assay buffer was then added and incubated for 60 min at room temperature. After the plate was washed three times with 200 μL of wash buffer, 50 μL of 3,3′,5,5′‐tetramethylbenzidine (SB02; Life Technologies, Guangzhou, China) was added and the plate was incubated in the dark for 20 min. After 25 μL of the stop solution was added (SS04; Life Technologies), the optical density (OD) of each well was measured within 10 min with a plate reader at 450 nm with a reference wavelength of 620 nm. All samples were tested in duplicate, and the specific binding ratio (SBR) was calculated using the following formula:

$$\text{SBR}=\frac{{\text{OD}}_{\mathrm{sample}}-{\text{OD}}_{\mathrm{NC}}}{{\text{OD}}_{\mathrm{PC}}-{\text{OD}}_{\mathrm{NC}}}.$$

### Development of B‐cell model

RPMI 1788 B‐lymphocyte cell line was purchased from the European Collection Authenticated Cell Cultures and cultured in Iscove's modified Dulbecco's medium supplemented with 10% FBS. Cultures were maintained between 3 × 10^5^ and 6 × 10^5^ cells·mL^−1^. Cells were grown at 37 °C and 5% CO_2_. To investigate the interactions between B cells and autoantigens of interest, RPMI 1788 cells were treated with 40 μg·mL^−1^ antigens (TSNARE1 or TRANK1) dissolved in DMSO or a vehicle control (DMSO treatment only). Cells were incubated at 37 °C and 5% CO_2_ for 48 h and then harvested for either analysis of cell surface marker CD83 or measurement of apoptotic cells.

### Analysis of CD83 B‐cell marker

Cultured cells were harvested as mentioned above, centrifuged at 150 ***g*** and washed with cold PBS. Pelleted cells were resuspended in 1% PBS–azide at a density of 1 × 10^7^ cells·mL^−1^, and 100 μL of cell suspension was then used for incubation with 10 μL anti‐human CD83‐APC (BD Bioscience, Oxford, UK) and another 100 μL for incubation with equimolar concentrations of isotype control, APC mouse IgG1 (BD Biosciences). All the samples were incubated on ice, in the dark for 20 min before being washed with PBS–azide followed by centrifugation at 150 ***g*** for 1 min. Pelleted cells were resuspended in 500 μL FACSFlow (BD Biosciences), and each sample was analysed by flow cytometry using the FACSCalibur within 15 min.

### Detection of apoptosis

Cultured cells were harvested as mentioned above, centrifuged at 150 ***g*** and washed with cold PBS. The cell pellet was resuspended in 1× Binding Buffer (Cat. No. 556547; BD Biosciences) to a concentration of 1 × 10^5^ cells·mL^−1^; 100 μL of cell suspension was transferred to an Eppendorf, and 5 μL Annexin V–FITC and 5 μL propidium iodide were added to each sample. Cells were gently vortexed and incubated for 15 min at room temperature in the dark. A 400 μL volume of 1× Binding Buffer was added to each tube and analysed by flow cytometry within 20 min.

### Data analysis

Shapiro–Wilk statistic was used to test a normal distribution of plasma IgG measurement data, but only one test showed a normal distribution (Table S2). Consequently, a Mann–Whitney *U*‐test was used to examine the differences in antibody levels between the patient group and the control group. To reduce the type I error due to multiple testing, a *P*‐value of < 0.007 was considered to be statistically significant as seven antigens were tested in this study. Receiver operating characteristic (ROC) curve analysis was performed on each of the antigens to calculate the area under the ROC curve (AUC) and the in‐house ELISA sensitivity against a specificity of ≥ 95%. ROC curve analysis is a plot of the true‐positive against the false‐positive rate, and the AUC represents a measure of how well each parameter is analysed to distinguish between the patient group and the control group. The coefficient of variation was used to represent an interassay deviation estimated using pooled plasma samples, called quality control sample, which were randomly collected from > 20 healthy subjects and tested on every 96‐well plate. Student's *t*‐test was used to examine the difference in the proportion of CD83 expression cells and the proportion of apoptotic cells, respectively.

## Results

Following exclusion of 33 cases based on change in diagnosis, a total of 181 patients aged 36.4 ± 13.2 years (88 males aged 34.1 ± 12.5 years and 93 females aged 38.7 ± 13.5 years) were included in the analyses. There were 227 healthy controls aged 36.8 ± 12.4 years (122 males and 105 females). The reproducibility of the in‐house ELISA test was acceptable, and all seven IgG tests had interassay deviation below 20% (Table S3).

Two of these seven tests showed a significant increase in plasma IgG levels between the patient group and the control group (Table 1), while there were significant differences between sexes. A significant increase in plasma IgG levels was found in patients with schizophrenia, including anti‐TSNARE1 IgG (*Z* = −4.332, *P* < 0.001) and anti‐CD25 IgG (*Z* = −3.756, *P* < 0.001); male patients mainly contributed to the increased levels of plasma anti‐TSNARE1 IgG (*Z* = −4.663, *P* < 0.001) and anti‐CD25 IgG (*Z* = −4.347, *P* < 0.001), whereas female patients failed to show any significant change in plasma IgG levels. All remaining results were not statistically significant (Table 1).

**Table 1:** Analysis of circulating IgG against individual antigens tested.

Antigen	Control, Mean ± SD	Patient, Mean ± SD	*Z*	*P* a
TSNARE1				
Male	0.587 ± 0.256	0.781 ± 0.312	−4.663	< 0.001
Female	0.716 ± 0.324	0.766 ± 0.327	−1.121	0.262
Both	0.647 ± 0.296	0.781 ± 0.323	−4.332	< 0.001
ZNF804A				
Male	0.675 ± 0.195	0.743 ± 0.210	−2.152	0.031
Female	0.716 ± 0.215	0.756 ± 0.229	−1.115	0.265
Both	0.694 ± 0.205	0.753 ± 0.222	−2.375	0.018
TRANK1				
Male	1.101 ± 0.415	1.185 ± 0.428	−1.391	0.164
Female	1.148 ± 0.434	1.233 ± 0.435	−1.120	0.263
Both	1.123 ± 0.424	1.214 ± 0.439	−1.870	0.061
DPYD				
Male	1.433 ± 0.603	1.463 ± 0.710	−0.045	0.964
Female	1.544 ± 0.619	1.591 ± 0.646	−0.507	0.612
Both	1.485 ± 0.611	1.525 ± 0.691	−0.434	0.664
TCF4				
Male	0.397 ± 0.123	0.405 ± 0.184	−0.579	0.563
Female	0.474 ± 0.171	0.417 ± 0.155	−2.244	0.025
Both	0.433 ± 0.152	0.413 ± 0.171	−1.489	0.136
CD25				
Male	0.731 ± 0.276	0.934 ± 0.438	−4.347	< 0.001
Female	0.833 ± 0.314	0.901 ± 0.429	−0.816	0.415
Both	0.779 ± 0.298	0.907 ± 0.391	−3.756	< 0.001
ERCC4				
Male	1.189 ± 0.615	1.417 ± 0.759	−1.849	0.064
Female	1.205 ± 0.535	1.281 ± 0.648	−0.538	0.590
Both	1.196 ± 0.578	1.326 ± 0.670	−1.702	0.089

^a^ *P* < 0.007 was considered statistically significant based on the Bonferroni correction as seven antigens were tested.

Receiver operating characteristic curve analysis revealed that of the seven IgG tests with a specificity of 95.2%, plasma anti‐TSNARE1 IgG assay had an AUC of 0.625 (95% CI 0.571–0.680) with a sensitivity of 15.7%, in which the IgG test in males showed an AUC of 0.689 with a sensitivity of 19.3% (95% CI 0.15–0.762); the remaining IgG tests all had a sensitivity of < 15% (Table 2).

**Table 2:** ROC analysis of IgG antibodies against individual autoantigens. SE, standard error.

Antigen	Sensitivity (%)^a^	AUC	SE	Asymptotic 95% CI	
				Lower	Upper
TSNARE1	15.7	0.625	0.028	0.571	0.680
Male	19.3	0.689	0.037	0.615	0.762
Female	14.4	0.547	0.041	0.466	0.628
ZNF804A	7.3	0.569	0.029	0.512	0.625
Male	11.4	0.587	0.040	0.509	0.665
Female	9.9	0.546	0.041	0.464	0.628
TRANK1	7.3	0.554	0.029	0.498	0.611
Male	8.0	0.556	0.041	0.477	0.636
Female	7.8	0.547	0.042	0.465	0.628
DPYD	6.7	0.513	0.029	0.455	0.570
Male	9.1	0.502	0.041	0.422	0.582
Female	6.7	0.521	0.041	0.439	0.603
TCF4	12.4	0.543	0.029	0.486	0.600
Male	13.6	0.523	0.043	0.440	0.607
Female	11.1	0.593	0.041	0.514	0.673
CD25	9.6	0.609	0.028	0.554	0.664
Male	12.5	0.676	0.037	0.603	0.749
Female	4.4	0.534	0.042	0.452	0.615
ERC4	10.1	0.549	0.029	0.493	0.606
Male	11.4	0.575	0.040	0.497	0.652
Female	8.9	0.522	0.042	0.441	0.604

^a ^Specificity of 95.2%.

**Figure 1:**
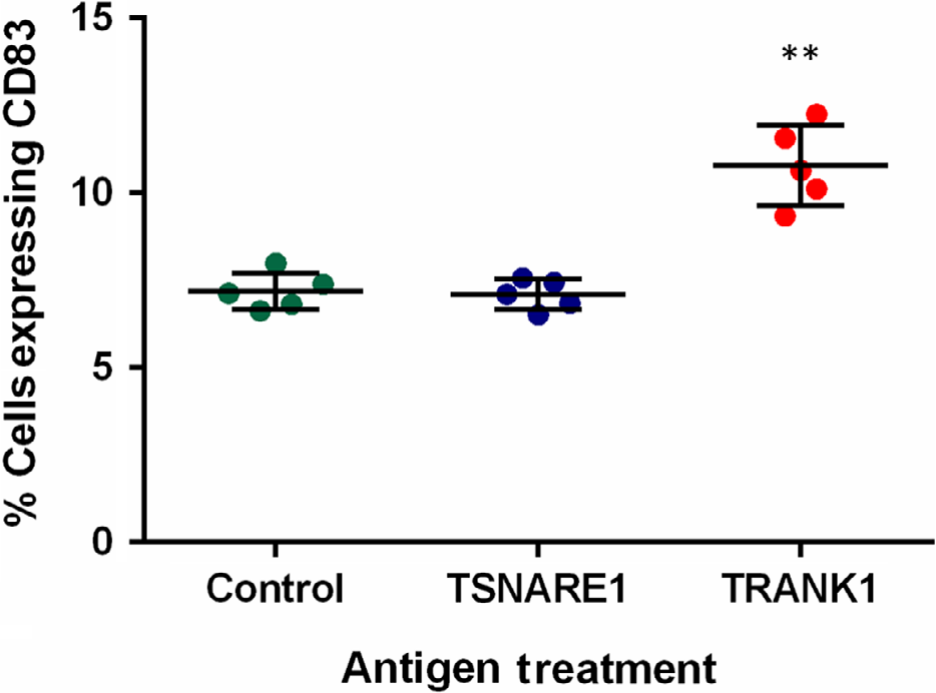
Changes in CD83+ cell proportion in RPMI B lymphocytes treated with 40 μg·mL^−1^ peptide antigens over 48 h. The error bars represent SD, and Student's *t*‐test was used to examine the difference in the proportion of CD83 expression cells; **P < 0.005 when compared with vehicle control. In this study, only *P* < 0.017 is considered statistically significant based on the Bonferroni correction as three independent treatments were given.

**Figure 2:**
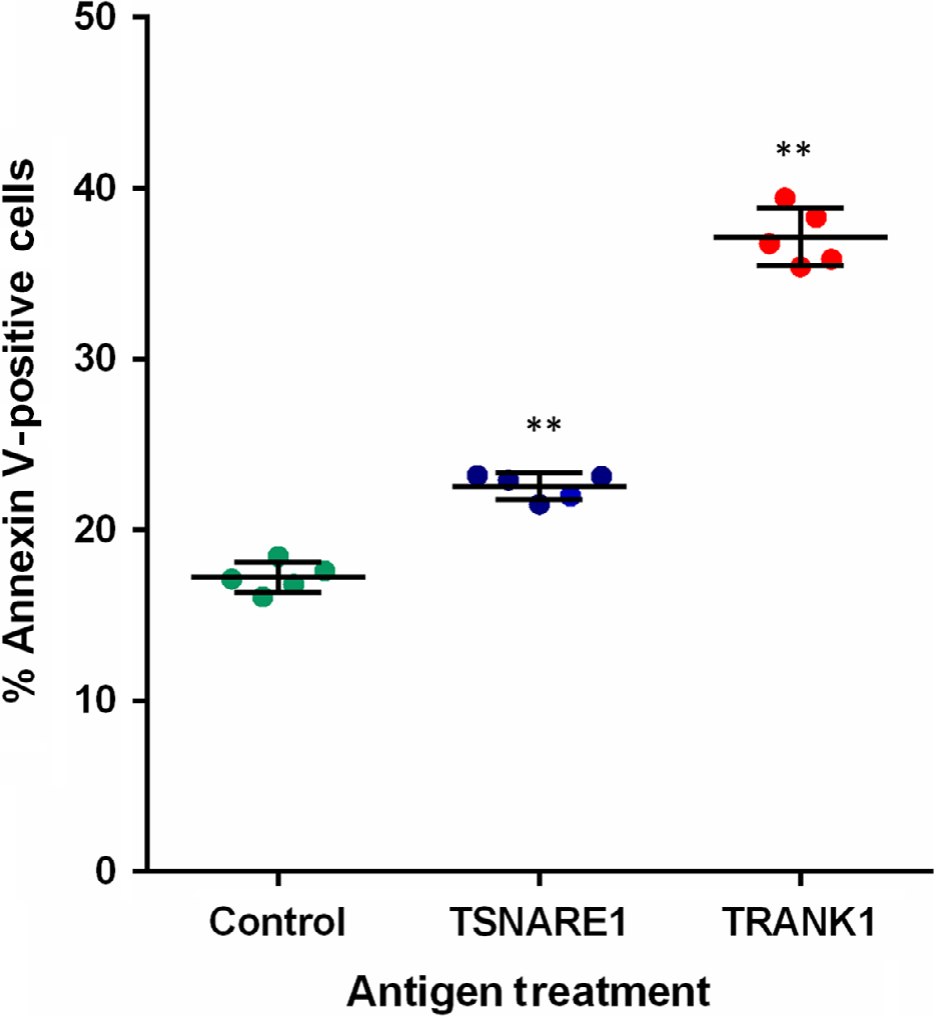
Analysis of apoptotic RPMI B lymphocytes treated with 40 μg·mL^−1^ peptide antigens over 48 h. The error bars represent SD, and Student's *t*‐test was used to examine the difference in the proportion of apoptotic cells; **P < 0.005 when compared with vehicle control. In this study, only *P* < 0.017 is considered statistically significant based on the Bonferroni correction as three independent treatments were given.

Work on the B‐cell model revealed that TRANK1‐derived antigen treatment was more likely to increase the proportion of CD83+ cells (Fig. 1) and apoptotic cells (Annexin V+) than TSNARE1‐derived antigen and vehicle treatments (Fig. 2).

## Discussion

The present study demonstrated that patients with schizophrenia had a significant increase in circulating anti‐TSNARE1 IgG levels, and male patients mainly contributed to the altered IgG levels. Altered antibodies for schizophrenia‐associated molecules are likely to be involved in a male population. It is worth noting that the anti‐TSNARE1 IgG assay has the highest sensitivity (true positive) of 15.7% (19.3% in males) against a specificity (true negative) of 95.2%, suggesting that anti‐TSNARE1 IgG may be indicative of a subgroup of schizophrenia.

The results of this study appear to be inconsistent with some from previous findings 16. In particular, our previous study showed that the anti‐TSNARE1 IgG levels had a significant decrease in individuals with chronic schizophrenia in comparison with healthy controls, whereas the present study demonstrated that individuals with schizophrenia had an increased anti‐TSNARE1 IgG level in plasma (Table 1). There are a number of possible reasons for this discrepancy. It is well established that there are significant differences in cytokine signalling in individuals with first‐episode psychosis as compared to both healthy controls and a chronically medicated population 11; the B‐cell model results suggest that B cells may be more tolerant to TSNARE1‐derived antigens than TRANK1‐derived antigens (Figs 1 and 2). Furthermore, our initial study was conducted in a Caucasian population and all patients received antipsychotic medication, whereas the present study was carried out in a Chinese population and all patients had first‐episode schizophrenia and were drug‐naïve. The inconsistent results should thus be attributable to the variation in subpopulations, medication, stage and duration of illness.

Gender differences in clinical presentation of schizophrenia have been well documented; male patients typically show earlier onset, greater severity and stronger resistance to antipsychotic medication than female patients 19, 20. The findings of this study suggest that the gender differences may arise as a result of immunological differences between males and females. Several studies have identified gender differences in autoimmune conditions among individuals with schizophrenia, showing that females had a higher incidence of autoimmune conditions than males 21, 22. Male patients were found to have higher levels of circulating autoantibodies against schizophrenia‐related targets than healthy controls, but female patients did not show a significant change in circulating autoantibody levels (Table 1). The gender differences in autoimmune responses should be taken into account for the development of precision treatment of schizophrenia.

The increased anti‐CD25 antibody levels may indicate a breakdown of immune tolerance in schizophrenia. CD25 is highly expressed in regulatory T cells that are involved in developing peripheral immune tolerance 23.

Response of B lymphocytes to 48‐h stimulation with TRANK1‐derived instead of TSNARE1‐derived peptide antigens could enhance the proportion of CD83+ cells and apoptotic B cells (Figs 1 and 2), suggesting that different schizophrenia‐related autoantigens have different effects on induction of central B‐cell tolerance, further supporting the role of immune tolerance breakdown in the development of schizophrenia. Once activated *in vivo*, B cells could initiate a number of cellular events including T‐cell activation and cytokine production. The increased antibody levels could result from a breakdown of immune tolerance to these schizophrenia‐associated proteins, causing an autoimmune response as discussed in our recent publication 16. However, increased natural IgG levels may reflect an increase in the expression of self‐molecules such as schizophrenia‐associated proteins due to genetic predisposition 15, leading to the activation of the class switch recombination; consequently, increased IgG may develop pathological immune responses such as autoimmune inflammation 24. Nevertheless, antibody studies in schizophrenia generally demonstrate inconsistencies, but this is largely a result of heterogeneity across studies.

There are a couple of limitations in this study. First, the tests conducted on the B‐lymphocyte cell line are limited due to the culture conditions that lack the required conditions to allow B‐cell maturation, resulting in increased apoptosis of peptide‐treated cells. If B cells collected from patients with schizophrenia were tested, a firm conclusion could be made. Second, it was almost impossible to control for lifestyle factors such as alcohol consumption, diets and use of any legal or illegal substances that may affect the antibody levels in the circulation. These confounding factors should be analysed in a future study that would be benefitted from the collection of confounding factors including BMI, smoking and drug use. It would be interesting to have access to clinical information for the participants including PANSS scores and mood evaluations; furthermore, access to other clinical biochemistry such as glucose would provide interesting insights. Finally, another limitation of this study is that female subjects were not well matched for age between the patient group and the control group, which may affect the levels of circulating antibodies tested. Future work should test a drug‐naïve Caucasian group and a medicated Chinese group to allow comparison of antibody measurements across populations.

## Conclusions

Several reports suggest that circulating autoantibodies for schizophrenia‐associated proteins may be indicative of an immunological subgroup of schizophrenia although the results reported to date have been inconclusive 25, 26. The state‐dependent alterations in cytokines may extend to a change in the whole immune system, and in some cases, immunomodulatory effects of antipsychotic medication may improve the efficacy in such a subgroup of schizophrenia patients. The activation of B lymphocytes by cognate autoantigens supports the role of genetic make‐up in tolerance breakdown and onset of abnormal autoimmunity in schizophrenia.

## Conflict of interest

The authors declare no conflict of interest.

## Author contributions

CL, RW and HY mainly carried out laboratory work, data analysis and drafting the manuscript; YJ, CQ and QM were mainly responsible for identification of patients and healthy controls, and collection of samples and clinical information; and JW conceived of this study, supervised laboratory work and data analysis, and corrected the manuscript.

## Supporting information


**Table S1.** Sequences of peptide antigens derived from target proteins tested.
**Table S2.** Shapiro‐Wilk test for a normal distribution of plasma IgG levels.
**Table S3.** Coefficient of variation (CV) for each antibody test with QC sample.
