# Supplementary material for: Anti‐TSNARE1 IgG plasma levels differ by sex in patients with schizophrenia in a Chinese population
Source: FEBS Open Bio. 2019 Sep 4;9(10):1705–12. doi: 10.1002/2211-5463.12704 (PMC6768289; doi:10.1002/2211-5463.12704)
Supplement: Supplementary file 1 — Table S1. Sequences of peptide antigens derived from target proteins tested. Table S2. Shapiro‐Wilk test for a normal distribution of plasma IgG levels. Table S3. Coefficient of variation (CV) for each antibody test with QC sample. [file FEB4-9-1705-s001.docx]

**Supplementary Information**

Table S1. Sequences of peptide antigens derived from target proteins tested

| Antigen | Sequence |
| --- | --- |
| DPYD | H-DIESILALN PRTQTH ATLCSTSAK KLD-OH |
| TSNARE1 | H-RCWLQE LFQEMSANVFRINSSVTSLER-OH |
| ZNF804A | H-ECYYIVISSTHLSNGHFRNIKGVFRGPLID-OH |
| TCF4 | H-CLKSDKPQTKLLILHQAVAVILSLEQQVRE-OH |
| TRANK1 | H-NILILSVRDARDWLM KTETRLKKEC-OH |
| ERCC4 | H-CQEAFILRLFRQKNKRGFIKAFTDNAVD-OH |
| CD25 | H-IYHFVVGQMVYYQCVQGYRALHRGPAESV-OH |

Table S2. Shapiro-Wilk test for a normal distribution of plasma IgG levels

|  | Control | | | | Case | | | | |
| --- | --- | --- | --- | --- | --- | --- | --- | --- | --- |
| Antigen | S | P | Skewness^a^ | Kurtosis^b^ | S | P | Skewness^c^ | | Kurtosis^d^ |
| TSNARE1  Male  Female | 0.932  0.942  0.930 | <0.001  0.011  <0.001 | 1.122  0.941  1.091 | 1.954  0.812  1.931 | 0.960  0.962  0.936 | <0.001  0.013  <0.001 | | 0.747  0.401  1.027 | 0.363  -0.651  1.162 |
| ZNF804A  Male  Female | 0.972  0.978  0.964 | 0.003  0.041  0.005 | 0.598  0.536  0.619 | 0.142  0.334  -0.097 | 0.987  0.976  0.988 | 0.122  0.107  0.597 | | 0.300  0.391  0.220 | -0.251  -0.236  -0.246 |
| TRANK1  Male  Female | 0.929  0.921  0.936 | <0.001  <0.001  <0.001 | 1.124  1.221  1.034 | 1.545  1.970  1.262 | 0.959  0.949  0.966 | <0.001  0.002  0.023 | | 0.708  0.791  0.641 | 0.194  0.356  0.147 |
| DPYD  Male  Female | 0.919  0.915  0.912 | <0.001  <0.001  <0.001 | 1.236  1.291  1.211 | 2.013  2.604  1.597 | 0.882  0.856  0.891 | <0.001  <0.001  <0.001 | | 1.679  1.905  1.504 | 4.506  5.826  3.491 |
| TCF4  Male  Female | 0.913  0.896  0.941 | <0.001  <0.001<0.001 | 1.440  1.635  1.140 | 3.737  5.041  2.715 | 0.891  0.859  0.908 | <0.001  <0.001  <0.001 | | 1.392  1.404  1.406 | 2.288  1.662  3.504 |
| CD25  Male  Female | 0.949  0.932  0.961 | <0.001  <0.0010.004 | 0.825  0.926  0.690 | 0.391  0.506  0.261 | 0.814  0.745  0.939 | <0.001  <0.001  <0.001 | | 2.841  3.371  0.973 | 17.165  19.190  1.092 |
| ERCC4  Male  Female | 0.889  0.871  0.908 | <0.001  <0.001  <0.001 | 1.673  1.851  1.375 | 5.185  6.188  3.203 | 0.862  0.827  0.923 | <0.001  <0.001  <0.001 | | 1.823  1.993  1.131 | 5.089  5.341  1.342 |

^a^ Standard error (SE) of 0.162 (males 0.219, females 0.236); ^b^ SE of 0.322 (males 0.435, females 0.467); ^c^ SE of 0.185 (males 0.261, females 0.217); ^d^ SE of 0.368 (males 0.517, females 0.431)

| Antigen | No of plates | Mean±SD | CV (%) |
| --- | --- | --- | --- |
| TSNARE1 | 21 | 0.895±0.165 | 18.44 |
| IL-6 | 21 | 3.352±0.548 | 16.35 |
| ZNF804A | 21 | 0.843±0.087 | 10.43 |
| TRANK1 | 21 | 1.350±0.196 | 14.52 |
| DPYD | 21 | 1.744±0.263 | 14.83 |
| TCF4 | 21 | 0.391±0.073 | 18.67 |
| CD25 | 21 | 0.912±0.114 | 12.50 |
| ERCC4 | 21 | 1.682±0.286 | 17.00 |

Table S3. Coefficient of variation (CV) for each antibody test with QC sample
